# Subacute large bowel obstruction as an unusual presentation of selective IgA deficiency

**DOI:** 10.1016/j.jacig.2026.100723

**Published:** 2026-04-30

**Authors:** Elizabeth Forouzanfar, Beena Nair, Pavaladurai Vijayadurai, Ariharan Anantharachagan

**Affiliations:** Lancashire Teaching Hospitals NHS Foundation Trust, Preston, United Kingdom

**Keywords:** Selective IgA deficiency, nodular lymphoid hyperplasia

## Abstract

Nodular lymphoid hyperplasia (NLH) is a rare, benign condition of lymphoid proliferation within the gastrointestinal tract. NLH is a recognized feature of specific immunodeficiency syndromes, including common variable immunodeficiency and selective IgA deficiency. NLH may develop throughout the gastrointestinal tract; however, it usually develops in the small intestine. We report an extremely unusual case of previously undiagnosed selective IgA deficiency presenting with subacute rectosigmoid obstruction.

## Case report

**Fig 1:**
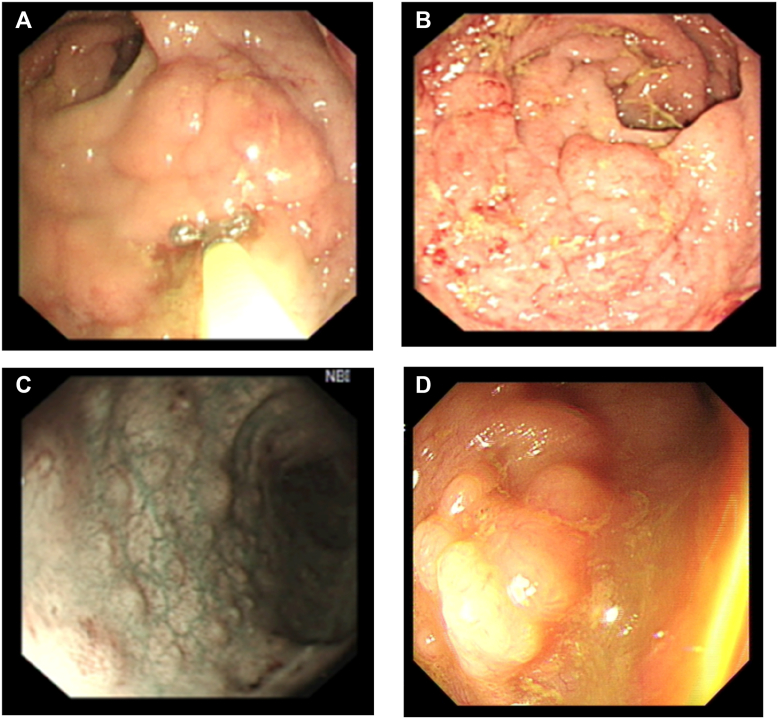
Colonoscopy image on the patient’s initial presentation with bowel obstruction. **A** and **B**, A nodular, circumferential, stenosing rectal mass with fungating appearance is demonstrated. **C,** Nodular appearance of the proximal sigmoid colon is evident. **D,** Diffuse nodular lymphoid hyperplasia of the terminal ileum is shown.

A gentleman in his mid-40s, with no previous medical history, developed progressive difficulty in opening his bowels over a 7-day period, culminating in complete bowel obstruction. Digital rectal examination identified an irregular, firm anorectal mass. The initial colonoscopy (Fig 1) showed a fungating rectal tumor, creating a high suspicion of malignancy. The rectum was severely edematous and stenosed, with diffuse mucosal changes throughout the entire rectum.

Computed tomography imaging indicated extensive rectosigmoid thickening extending approximately 11 cm, breaching into serosa with significant perirectal stranding. Mild splenomegaly (16.7 cm craniocaudally) was noted.

**Fig 2:**
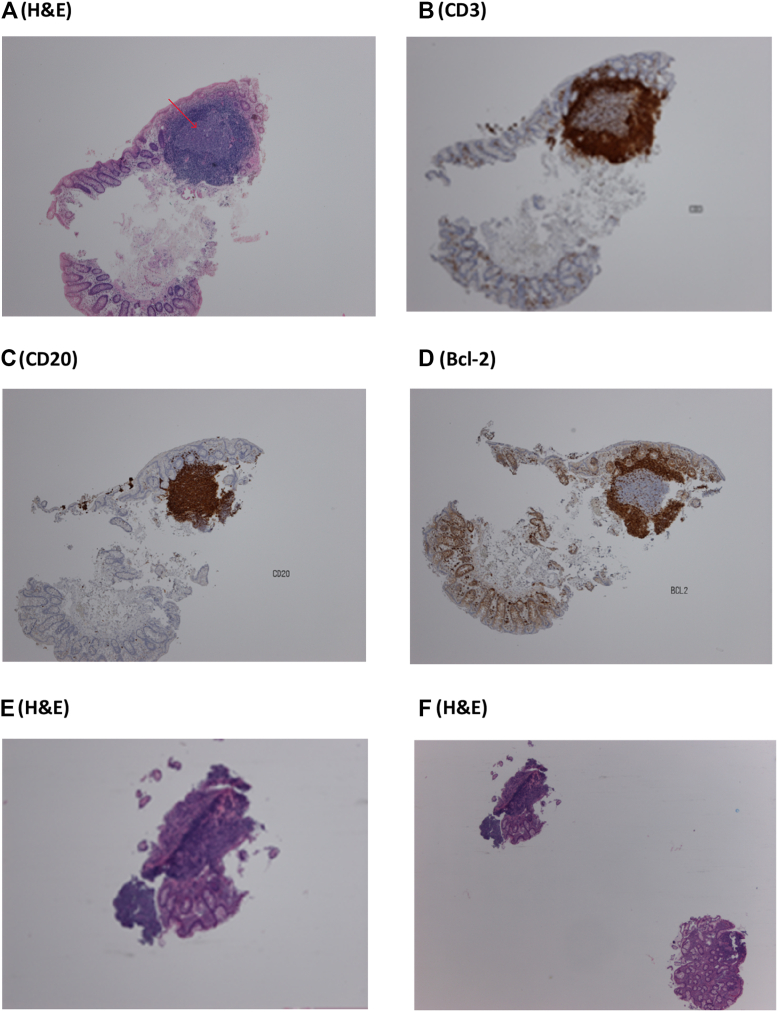
Initial histology of the rectal mucosa. **A-D,** A lymphoid nodule with a germinal center (*arrow*) is visible. Immunohistochemistry (*brown stain*) identified a mixed population of B and T cells. The results of staining for Bcl-2, CD5, and CD10 were negative. Extensive molecular analysis failed to identify a clonal B-cell population. Repeat biopsy following corticosteroid therapy (**E** and **F**) identified a focal lymphoid aggregate with no evidence of malignancy. *H&E*, Hemotoxylin and eosin.

Initial histologic examination (Fig 2) demonstrated a dense lymphoid infiltrate, predominantly composed of B cells, that was concerning for lymphoproliferation. Fragmented and irregular remnants of reactive germinal centers were visualized. Molecular testing was unable to demonstrate a clonal B-cell population, as a result of which no diagnosis of malignancy could be made. DNA amplification demonstrated a polyclonal pattern. Repeat biopsy revealed similar features, and no malignancy could be confirmed. A third biopsy confirmed lymphoid hyperplasia rather than malignancy. At that time, the patient underwent a gastroduodenoscopy, which identified no macroscopic abnormality on visual examination of the upper gastrointestinal tract (GIT). The findings of esophageal, gastric, and duodenal biopsies from this study were within normal limits. A bone marrow biopsy was then performed; it did not demonstrate evidence of hematologic malignancy.

Finally, testing for serum immunoglobulins was requested. Serum IgA was undetectable (<0.05 g/L) in the context of normal serum levels of IgG. IgM level was also borderline low (0.39g/L [normal range 0.50-2.00g/L]), possibly because this patient had commenced taking high-dose prednisolone 4 months prior. No alternative etiology was identified. Immunophenotypic analysis demonstrated B-cell counts to be within normal limits. The results of serum-free light chain analysis and serum electrophoresis were unremarkable. The patient had no features of allergy, and there was no history of recurrent bacterial sinopulmonary infections or autoimmune disease.

High-dose oral corticosteroid therapy was eventually commenced (60 mg of prednisolone once daily) after discussion with the immunology department, resulting in complete symptom resolution over 14 days. This prednisolone dose was gradually reduced to a maintenance dose of 10 mg once daily. Oral prednisolone therapy was inadvertently continued for 12 years at a dose of 10 mg once daily, as the patient was lost to follow-up by the immunology clinic.

Repeat colonoscopy 12 years later demonstrated mild lymphoid hyperplasia at the ileocecal valve; however, the rest of the colon appeared macroscopically normal. The patient was asymptomatic at that time. The patient has discontinued steroid therapy, and no symptom recurrence has been reported to date.

## Discussion

Selective IgA deficiency (SIgAD) is the most common immune defect, with a prevalence ranging from 1 in 3000 to 1 in 550, with wide variation between ethnic groups.^1^ It is defined as a serum IgA level less than 0.05 g/L in the presence of normal concentrations of both IgG and IgM in patients older than 4 years, with no alternative cause identified.^2^ The majority of individuals are asymptomatic, and diagnoses are often made incidentally during other investigations (eg, for celiac disease). IgA-deficient individuals are recognized to be at increased risk of allergic and autoimmune disease, recurrent sinopulmonary infections, and transfusion reactions owing to formation of anti-IgA antibodies.

Nodular lymphoid hyperplasia (NLH) is a rare condition characterized by proliferation of lymphoid tissue within the GIT.^3^ Its etiology is unknown, and it has been identified in all age groups. Hypotheses as to the mechanism of NLH have centred around a localized, adaptive response to a pathogen; several bacteria, including *Helicobacter pylori* and *Giardia lamblia,* have been considered.

NLH, particularly that presenting in adults, should prompt investigation into an occult immune defect. SIgAD and common variable immunodeficiency are associated with NLH.^3^

NLH can be identified incidentally in asymptomatic patients; however, depending on the site and extent of disease, it can cause abdominal pain, diarrhea, and bleeding. Very rarely, NLH can present with intestinal obstruction of the small bowel or intussusception. This is more common in children. Diagnosis is generally achieved on endoscopy. Typical features include the presence of multiple nodules ranging in size from 2 to 10 mm. The terminal ileum is the most commonly affected site.^4^ Histologic assessment is therefore required to confirm the diagnosis. Hyperplastic lymphoid follicles and increased mitotic activity within germinal centers is consistent with a histologic diagnosis of NLH.

This case demonstrates that NLH secondary to SIgAD can affect distal portions of the GIT, including the rectum and sigmoid colon. To our knowledge, this is the first adult case in the literature to describe sigmoid colonic obstruction secondary to NLH.

Ethical approval statement: This case report represents a detailed report of an individual patient identified during routine diagnostics and does not belong to a larger project for which ethics committee approval would be required. Ethical approval was therefore not sought for this case report.

Patient and public involvement: The patient described in this case report gave full consent to publication of this case report, including the accompanying images. The patient reviewed the article and images before submission. No further patient and public involvement was considered for this project.

## Disclosure statement

Disclosure of potential conflict of interest: The authors declare that they have no relevant conflicts of interest.
